# Crystal structure of poly[μ-acetato-bis­[μ-2-oxo-2-(quinolin-8-yl)ethano­ato]tris­odium]

**DOI:** 10.1107/S1600536814023423

**Published:** 2014-10-31

**Authors:** Rachel L. Nicholls, Christopher M. Pask, Bao Nguyen

**Affiliations:** a School of Chemistry, University of Leeds, Woodhouse Lane, Leeds, LS2 9JT, England

**Keywords:** crystal structure, keto acid, sodium, acetate

## Abstract

The title compound [Na_3_(C_11_H_6_NO_3_)_2_(C_2_H_3_O_2_)]_*n*_, crystallized through diffusion of diethyl ether into methanol as needles. There are three crystallographically independent Na^+^ cations present, each exhib­it­ing a distorted octa­hedral coordination geometry, two through coordination by five O atoms and one N atom, and one through coordination by six O atoms. A series of inter­molecular O⋯Na and N⋯Na contacts leads to the formation of chains along the *a-*axis direction.

## Related literature   

The sodium salt of 2-oxo-2-(quinolin-8-yl)ethanoic acid was prepared as an authentic product during a catalytic process development within our group. Ethyl 2-oxo-2-(quinolin-8-yl)ethano­ate was prepared by a literature procedure (Crespo-Peña *et al.*, 2012 ▶) and then hydrolysed under basic conditions to yield the title compound. For sodium salts of keto-acids, see; Lis & Matuszewski (1984 ▶); Jain *et al.* (1969 ▶); Tavale *et al.* (1961 ▶, 1964 ▶); Rach *et al.* (1988 ▶). A similar Na⋯C=N(quinoline) inter­action is observed in a previously published samarium Schiff base complex (Li *et al.*, 2008 ▶). 
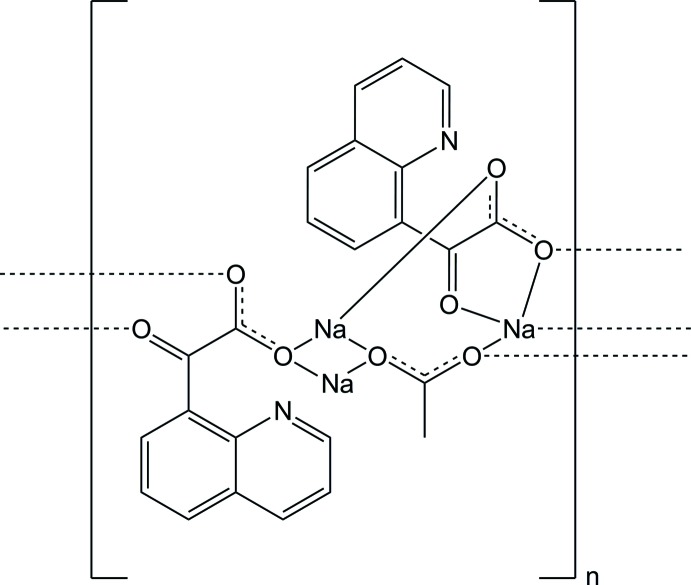


## Experimental   

### Crystal data  

[Na_3_(C_11_H_6_NO_3_)_2_(C_2_H_3_O_2_)]
*M* *_r_* = 528.35
Monoclinic, 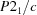

*a* = 6.1101 (5) Å
*b* = 22.7075 (19) Å
*c* = 16.1587 (12) Å
β = 94.626 (7)°
*V* = 2234.6 (3) Å^3^
*Z* = 4
Cu *K*α radiation
μ = 1.50 mm^−1^
*T* = 120 K
0.19 × 0.04 × 0.03 mm

### Data collection  

Agilent SuperNova (Dual, Cu at zero, Atlas) diffractometer
Absorption correction: analytical [*CrysAlis PRO* (Agilent, 2014 ▶), based on expressions derived by Clark & Reid (1995 ▶)] *T*_min_ = 0.887, *T*_max_ = 0.971
7799 measured reflections
3943 independent reflections
2557 reflections with *I* > 2σ(*I*)
*R*_int_ = 0.059

### Refinement  

*R*[*F* ^2^ > 2σ(*F* ^2^)] = 0.050
*wR*(*F* ^2^) = 0.130
*S* = 0.99
3943 reflections
335 parameters
H-atom parameters constrained
Δρ_max_ = 0.25 e Å^−3^
Δρ_min_ = −0.29 e Å^−3^

### 

Data collection: *CrysAlis PRO* (Agilent, 2014 ▶); cell refinement: *CrysAlis PRO*; data reduction: *CrysAlis PRO*; program(s) used to solve structure: *SHELXS97* (Sheldrick, 2008 ▶); program(s) used to refine structure: *SHELXL97* (Sheldrick, 2008 ▶); molecular graphics: *OLEX2* (Dolomanov *et al.*, 2009 ▶); software used to prepare material for publication: *publCIF* (Westrip, 2010 ▶).

## Supplementary Material

Crystal structure: contains datablock(s) I. DOI: 10.1107/S1600536814023423/pj2016sup1.cif

Structure factors: contains datablock(s) I. DOI: 10.1107/S1600536814023423/pj2016Isup2.hkl

**Fig. 1.**
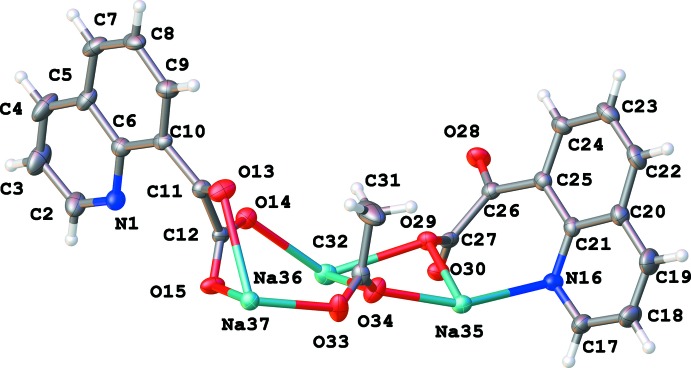
The asymmetric unit of (1) showing the labelling scheme. Displacement ellipsoids are at the 50% probability level. Hydrogen atoms have been omitted for clarity.

**Fig. 2.**
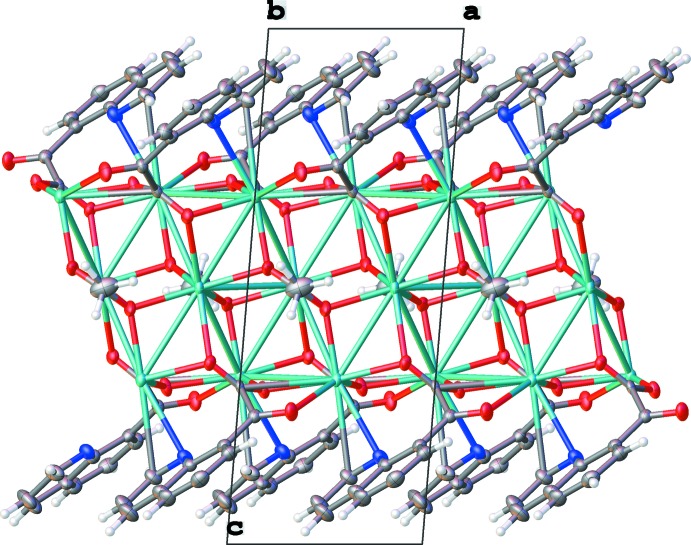
Partial packing diagram of (1) showing the one-dimensional chain along the crystallographic a-axis. Displacement ellipsoids are at the 50% probability level.

**Fig. 3.**
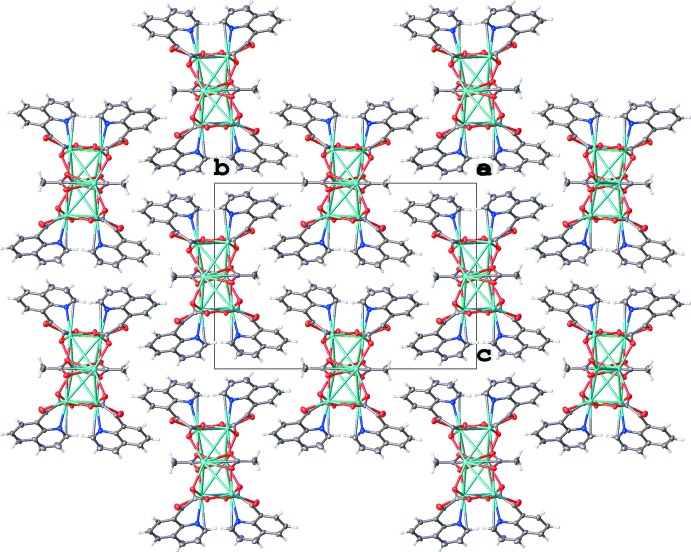
Partial packing diagram of (1) viewed on the bc plane. Displacement ellipsoids are at the 50% probability level.

. DOI: 10.1107/S1600536814023423/pj2016fig1.tif
The asymmetric unit of (1) showing the labelling scheme. Displacement ellipsoids are at the 50% probability level. Hydrogen atoms have been omitted for clarity.

a . DOI: 10.1107/S1600536814023423/pj2016fig2.tif
Partial packing diagram of (1) showing the one-dimensional chain along the crystallographic *a*-axis. Displacement ellipsoids are at the 50% probability level.

bc . DOI: 10.1107/S1600536814023423/pj2016fig3.tif
Partial packing diagram of (1) viewed on the *bc* plane. Displacement ellipsoids are at the 50% probability level.

CCDC reference: 1030741

Additional supporting information:  crystallographic information; 3D view; checkCIF report

## supplementary crystallographic information

### S1. Comment

The sodium salt (1) of 2-oxo-2-(quinolin-8-yl)ethanoic acid was prepared as an authentic product during a catalytic process development within our group. Ethyl 2-oxo-2-(quinolin-8-yl)ethano­ate was prepared by a literature procedure (Crespo-Peña *et al.*, 2012) and then hydrolysed under basic conditions to yield the title compound.

The asymmetric unit of 1 (Fig. 1) contains two crystallographically independent 2-oxo-2-(quinolin-8-yl)ethano­ate anions, one acetate anion and three crystallographically independent sodium cations. Each sodium cation exhibits distorted o­cta­hedral geometry. One sodium cation (Na36) is coordinated by six oxygen atoms from the oxo-2'-quinolin-8'-yl-ethano­ate and acetate ions. Na···O bond distances are in the range 2.290 (3) to 2.610 (3) Å. The other two sodium cations (Na35, Na37) are coordinated by five oxygen atoms (2.272 (3)-2.727 (3) Å) and what appears to be an η_2_ inter­action with a C=N of the quinoline ring (Na···N = 2.769 (3), 2.814 (3); Na···C = 3.035 (4), 3.073 (4)). A similar Na···C=N (quinoline) inter­action is observed in a previously published samarium Schiff base complex (Li *et al.*, 2008), although this is somewhat shorter than that observed in 1. Na···O bond lengths are comparable to previously published sodium salts of keto acids (Lis *et al.*, 1984, Jain *et al.*, 1969, Tavale *et al.*, 1961, 1964, Rach *et al.*, 1988).

These Na···O inter­actions lead to the formation of one-dimensional chains along the crystallographic *a*-axis (Fig. 2). The quinoline rings of the oxo(8-quinolyl)acetate groups and methyl groups of acetate ions appear to act as a hydro­phobic sheath, encapsulating the Na···O core of the chains, keeping them separated in the solid state. (Fig. 3).

### S2. Experimental

A solution of ethyl 2-oxo-2-(quinolin-8-yl)ethano­ate (217 mg, 0.947 mmol) in methanol (3mL) was cooled to 0 °C and aqueous 2M NaOH (3 mL) added and stirred at room temperature for 90 min. The methanol was removed under vacuum and the remaining aqueous solution was washed at pH 9 with ethyl acetate (2 x 20 mL). The aqueous solution was acidified to pH 5 with 1M HCl and washed with ethyl acetate (2 x 20 mL). The remaining aqueous solution was reduced under vacuum to give a white solid (0.231 g) which was dissolved in methanol and filtered to remove NaCl. The product was recrystallised through diffusion of di­ethyl ether into a solution of the product in a minimum amount of methanol over three days to afford colourless needles.

### S3. Refinement

No special refinement procedures were applied to this crystal structure. All hydrogen atoms were placed in calculated positions and refined isotropically using a riding model.

### Crystal data

**Table:**

[Na_3_(C_11_H_6_NO_3_)_2_(C_2_H_3_O_2_)]	*F*(000) = 1080
*M**_r_* = 528.35	*D*_x_ = 1.570 Mg m^−^^3^
Monoclinic, *P*2_1_/*c*	Cu *K*α radiation, λ = 1.54184 Å
*a* = 6.1101 (5) Å	Cell parameters from 1524 reflections
*b* = 22.7075 (19) Å	θ = 5.5–72.4°
*c* = 16.1587 (12) Å	µ = 1.50 mm^−^^1^
β = 94.626 (7)°	*T* = 120 K
*V* = 2234.6 (3) Å^3^	Needle, colourless
*Z* = 4	0.19 × 0.04 × 0.03 mm

### Data collection

**Table:**

Agilent SuperNova (Dual, Cu at zero, Atlas) diffractometer	3943 independent reflections
Radiation source: sealed X-ray tube, SuperNova (Cu) X-ray Source	2557 reflections with *I* > 2σ(*I*)
Mirror monochromator	*R*_int_ = 0.059
Detector resolution: 10.6191 pixels mm^-1^	θ_max_ = 66.6°, θ_min_ = 3.4°
ω scans	*h* = −4→7
Absorption correction: analytical [*CrysAlis PRO* (Agilent, 2014), based on expressions derived by Clark & Reid (1995)]	*k* = −25→26
*T*_min_ = 0.887, *T*_max_ = 0.971	*l* = −18→19
7799 measured reflections	

### Refinement

**Table:**

Refinement on *F*^2^	Primary atom site location: structure-invariant direct methods
Least-squares matrix: full	Secondary atom site location: difference Fourier map
*R*[*F*^2^ > 2σ(*F*^2^)] = 0.050	Hydrogen site location: inferred from neighbouring sites
*wR*(*F*^2^) = 0.130	H-atom parameters constrained
*S* = 0.99	*w* = 1/[σ^2^(*F*_o_^2^) + (0.0475*P*)^2^] where *P* = (*F*_o_^2^ + 2*F*_c_^2^)/3
3943 reflections	(Δ/σ)_max_ < 0.001
335 parameters	Δρ_max_ = 0.25 e Å^−^^3^
0 restraints	Δρ_min_ = −0.29 e Å^−^^3^

### Special details

**Table:**

Experimental. Absorption correction: CrysAlisPro, Agilent Technologies, Version 1.171.37.33 (release 27-03-2014 CrysAlis171 .NET) (compiled Mar 27 2014,17:12:48) Analytical numeric absorption correction using a multifaceted crystal model based on expressions derived by R.C. Clark & J.S. Reid. (Clark, R. C. & Reid, J. S. (1995). Acta Cryst. A51, 887-897) Empirical absorption correction using spherical harmonics, implemented in SCALE3 ABSPACK scaling algorithm.
Geometry. All e.s.d.'s (except the e.s.d. in the dihedral angle between two l.s. planes) are estimated using the full covariance matrix. The cell e.s.d.'s are taken into account individually in the estimation of e.s.d.'s in distances, angles and torsion angles; correlations between e.s.d.'s in cell parameters are only used when they are defined by crystal symmetry. An approximate (isotropic) treatment of cell e.s.d.'s is used for estimating e.s.d.'s involving l.s. planes.
Refinement. Refinement of *F*^2^ against ALL reflections. The weighted *R*-factor *wR* and goodness of fit *S* are based on *F*^2^, conventional *R*-factors *R* are based on *F*, with *F* set to zero for negative *F*^2^. The threshold expression of *F*^2^ > σ(*F*^2^) is used only for calculating *R*-factors(gt) *etc*. and is not relevant to the choice of reflections for refinement. *R*-factors based on *F*^2^ are statistically about twice as large as those based on *F*, and *R*- factors based on ALL data will be even larger.

### Fractional atomic coordinates and isotropic or equivalent isotropic displacement parameters (Å^2^)

**Table:**

	*x*	*y*	*z*	*U*_iso_*/*U*_eq_	
N1	0.2460 (5)	0.59015 (12)	0.32073 (16)	0.0248 (6)	
C2	0.0893 (6)	0.56634 (17)	0.3616 (2)	0.0324 (8)	
H2	0.0647	0.5261	0.3557	0.039*	
C3	−0.0423 (7)	0.59804 (19)	0.4134 (2)	0.0447 (11)	
H3	−0.1491	0.5789	0.4413	0.054*	
C4	−0.0117 (7)	0.65702 (19)	0.4222 (2)	0.0428 (10)	
H4	−0.0980	0.6786	0.4561	0.051*	
C5	0.1503 (5)	0.68536 (16)	0.3801 (2)	0.0267 (7)	
C6	0.2792 (5)	0.65001 (14)	0.33027 (19)	0.0220 (7)	
C7	0.1939 (6)	0.74641 (16)	0.3868 (2)	0.0303 (8)	
H7	0.1080	0.7698	0.4186	0.036*	
C8	0.3585 (6)	0.77153 (15)	0.3477 (2)	0.0281 (8)	
H8	0.3841	0.8118	0.3524	0.034*	
C9	0.4899 (6)	0.73642 (14)	0.3001 (2)	0.0259 (7)	
H9	0.6058	0.7538	0.2752	0.031*	
C10	0.4523 (5)	0.67691 (13)	0.28926 (18)	0.0197 (6)	
C11	0.5912 (5)	0.64329 (14)	0.23363 (19)	0.0204 (7)	
C12	0.5049 (5)	0.58940 (13)	0.18503 (18)	0.0180 (6)	
O13	0.7770 (4)	0.66025 (11)	0.22217 (15)	0.0303 (6)	
O14	0.3382 (4)	0.59685 (10)	0.13640 (13)	0.0237 (5)	
O15	0.6225 (4)	0.54440 (10)	0.19491 (14)	0.0255 (5)	
N16	0.2494 (4)	0.56492 (12)	−0.33221 (16)	0.0245 (6)	
C17	0.4040 (6)	0.53700 (16)	−0.3695 (2)	0.0285 (8)	
H17	0.4232	0.4970	−0.3590	0.034*	
C18	0.5401 (7)	0.56398 (17)	−0.4235 (2)	0.0371 (9)	
H18	0.6454	0.5422	−0.4487	0.045*	
C19	0.5161 (7)	0.62277 (17)	−0.4390 (2)	0.0385 (9)	
H19	0.6037	0.6413	−0.4755	0.046*	
C20	0.3589 (6)	0.65517 (15)	−0.3995 (2)	0.0268 (7)	
C21	0.2230 (5)	0.62401 (14)	−0.34723 (18)	0.0200 (6)	
C22	0.3271 (6)	0.71665 (16)	−0.4110 (2)	0.0310 (8)	
H22	0.4179	0.7375	−0.4440	0.037*	
C23	0.1646 (6)	0.74553 (16)	−0.3741 (2)	0.0307 (8)	
H23	0.1461	0.7859	−0.3816	0.037*	
C24	0.0249 (5)	0.71412 (14)	−0.32455 (19)	0.0241 (7)	
H24	−0.0893	0.7338	−0.3017	0.029*	
C25	0.0544 (5)	0.65465 (14)	−0.30924 (18)	0.0208 (7)	
C26	−0.0895 (5)	0.62462 (13)	−0.25169 (18)	0.0200 (6)	
C27	−0.0049 (5)	0.57333 (13)	−0.19636 (19)	0.0182 (6)	
O28	−0.2754 (4)	0.64243 (11)	−0.24306 (15)	0.0288 (5)	
O29	0.1611 (3)	0.58346 (10)	−0.14860 (13)	0.0219 (5)	
O30	−0.1234 (3)	0.52831 (10)	−0.20085 (13)	0.0232 (5)	
C31	0.7331 (9)	0.65337 (18)	−0.0025 (3)	0.0526 (12)	
H31A	0.5884	0.6654	0.0096	0.079*	
H31B	0.7644	0.6685	−0.0557	0.079*	
H31C	0.8387	0.6684	0.0395	0.079*	
C32	0.7452 (5)	0.58759 (14)	−0.00364 (19)	0.0205 (7)	
O33	0.9040 (4)	0.56211 (12)	0.03523 (14)	0.0312 (6)	
O34	0.5933 (4)	0.55990 (11)	−0.04300 (14)	0.0278 (5)	
Na35	0.50542 (19)	0.54983 (5)	−0.18100 (7)	0.0201 (3)	
Na36	0.2478 (2)	0.54062 (6)	−0.00310 (8)	0.0247 (3)	
Na37	0.99681 (19)	0.56515 (5)	0.17420 (7)	0.0198 (3)	

### Atomic displacement parameters (Å^2^)

**Table:**

	*U*^11^	*U*^22^	*U*^33^	*U*^12^	*U*^13^	*U*^23^
N1	0.0263 (16)	0.0231 (15)	0.0257 (13)	−0.0049 (11)	0.0074 (11)	−0.0003 (12)
C2	0.041 (2)	0.0288 (19)	0.0285 (17)	−0.0146 (15)	0.0098 (15)	−0.0036 (15)
C3	0.044 (2)	0.053 (3)	0.041 (2)	−0.020 (2)	0.0246 (18)	−0.013 (2)
C4	0.041 (2)	0.049 (3)	0.041 (2)	−0.0106 (18)	0.0226 (18)	−0.0217 (19)
C5	0.0249 (19)	0.0326 (19)	0.0230 (16)	−0.0020 (14)	0.0042 (13)	−0.0095 (14)
C6	0.0215 (17)	0.0213 (17)	0.0228 (15)	−0.0020 (12)	0.0001 (12)	−0.0012 (13)
C7	0.0286 (19)	0.0312 (19)	0.0310 (18)	0.0072 (14)	0.0005 (14)	−0.0131 (15)
C8	0.040 (2)	0.0166 (16)	0.0269 (16)	0.0015 (14)	−0.0046 (15)	−0.0049 (14)
C9	0.0260 (18)	0.0201 (17)	0.0314 (17)	−0.0006 (13)	0.0007 (13)	−0.0020 (14)
C10	0.0200 (17)	0.0187 (16)	0.0199 (14)	−0.0002 (12)	−0.0030 (12)	−0.0037 (13)
C11	0.0175 (17)	0.0204 (16)	0.0237 (15)	−0.0006 (12)	0.0043 (12)	−0.0002 (13)
C12	0.0168 (16)	0.0168 (16)	0.0215 (14)	−0.0002 (12)	0.0080 (12)	0.0007 (12)
O13	0.0193 (13)	0.0291 (13)	0.0431 (13)	−0.0082 (9)	0.0065 (10)	−0.0104 (11)
O14	0.0174 (12)	0.0270 (12)	0.0267 (11)	−0.0040 (9)	0.0018 (9)	−0.0018 (10)
O15	0.0183 (12)	0.0200 (12)	0.0395 (13)	0.0009 (9)	0.0108 (10)	−0.0027 (10)
N16	0.0279 (16)	0.0189 (14)	0.0277 (14)	0.0010 (11)	0.0087 (11)	0.0011 (12)
C17	0.034 (2)	0.0255 (18)	0.0274 (16)	0.0027 (14)	0.0107 (14)	−0.0026 (14)
C18	0.041 (2)	0.034 (2)	0.0386 (19)	0.0038 (17)	0.0206 (17)	−0.0013 (17)
C19	0.045 (2)	0.040 (2)	0.0339 (19)	−0.0035 (17)	0.0211 (17)	0.0055 (18)
C20	0.0318 (19)	0.0251 (18)	0.0242 (15)	−0.0025 (14)	0.0059 (14)	0.0033 (14)
C21	0.0219 (17)	0.0211 (16)	0.0170 (14)	−0.0043 (12)	0.0012 (12)	0.0001 (12)
C22	0.035 (2)	0.030 (2)	0.0284 (17)	−0.0062 (15)	0.0047 (14)	0.0106 (15)
C23	0.036 (2)	0.0226 (18)	0.0320 (18)	−0.0030 (14)	−0.0043 (15)	0.0071 (15)
C24	0.0265 (18)	0.0204 (17)	0.0246 (16)	0.0025 (13)	−0.0027 (13)	0.0019 (13)
C25	0.0213 (17)	0.0215 (17)	0.0193 (14)	−0.0005 (12)	−0.0008 (12)	0.0008 (12)
C26	0.0201 (17)	0.0190 (16)	0.0206 (14)	0.0004 (12)	0.0007 (12)	−0.0029 (13)
C27	0.0133 (16)	0.0181 (16)	0.0245 (15)	0.0017 (11)	0.0085 (12)	−0.0015 (13)
O28	0.0186 (13)	0.0308 (13)	0.0375 (13)	0.0084 (10)	0.0045 (10)	0.0062 (11)
O29	0.0153 (12)	0.0242 (12)	0.0258 (11)	0.0035 (8)	−0.0010 (9)	0.0005 (9)
O30	0.0169 (12)	0.0212 (12)	0.0323 (12)	−0.0004 (9)	0.0070 (9)	0.0016 (10)
C31	0.084 (3)	0.025 (2)	0.046 (2)	−0.007 (2)	−0.013 (2)	0.0025 (18)
C32	0.0135 (15)	0.0274 (18)	0.0205 (14)	−0.0018 (12)	0.0004 (12)	0.0025 (13)
O33	0.0159 (12)	0.0532 (16)	0.0243 (11)	0.0085 (10)	0.0012 (9)	0.0068 (11)
O34	0.0150 (12)	0.0415 (15)	0.0269 (11)	−0.0051 (9)	0.0013 (9)	−0.0098 (11)
Na35	0.0146 (6)	0.0211 (6)	0.0245 (6)	0.0003 (4)	0.0016 (5)	−0.0015 (5)
Na36	0.0124 (6)	0.0279 (7)	0.0341 (7)	0.0005 (5)	0.0035 (5)	0.0016 (5)
Na37	0.0149 (6)	0.0221 (6)	0.0224 (6)	−0.0002 (4)	0.0019 (4)	0.0006 (5)

### Geometric parameters (Å, º)

**Table:**

N1—C2	1.321 (4)	C24—H24	0.9300
N1—C6	1.381 (4)	C24—C25	1.382 (5)
N1—Na37^i^	2.769 (3)	C25—C26	1.495 (4)
C2—H2	0.9300	C26—C27	1.532 (4)
C2—C3	1.406 (5)	C26—O28	1.224 (4)
C2—Na37^i^	3.035 (4)	C27—O29	1.245 (4)
C3—H3	0.9300	C27—O30	1.252 (4)
C3—C4	1.358 (6)	C27—Na35^i^	3.069 (3)
C4—H4	0.9300	O28—Na35^i^	2.727 (3)
C4—C5	1.402 (5)	O29—Na35	2.337 (2)
C5—C6	1.419 (5)	O29—Na36	2.560 (2)
C5—C7	1.414 (5)	O30—Na35^i^	2.367 (2)
C6—C10	1.430 (5)	O30—Na37^ii^	2.288 (2)
C7—H7	0.9300	C31—H31A	0.9600
C7—C8	1.355 (5)	C31—H31B	0.9600
C8—H8	0.9300	C31—H31C	0.9600
C8—C9	1.404 (5)	C31—C32	1.496 (5)
C9—H9	0.9300	C32—O33	1.254 (4)
C9—C10	1.380 (5)	C32—O34	1.251 (4)
C10—C11	1.495 (4)	C32—Na36^ii^	2.914 (4)
C11—C12	1.525 (4)	O33—Na36^ii^	2.549 (3)
C11—O13	1.227 (4)	O33—Na36^iii^	2.290 (3)
C12—O14	1.247 (4)	O33—Na37	2.272 (2)
C12—O15	1.252 (4)	O34—Na35	2.263 (2)
C12—Na37	3.074 (3)	O34—Na36	2.299 (2)
O13—Na37	2.690 (3)	O34—Na36^ii^	2.567 (3)
O14—Na36	2.610 (3)	Na35—O15^ii^	2.283 (2)
O14—Na37^i^	2.334 (2)	Na35—C27^iii^	3.069 (3)
O15—Na35^ii^	2.283 (2)	Na35—O28^iii^	2.727 (3)
O15—Na37	2.385 (2)	Na35—O30^iii^	2.367 (2)
N16—C17	1.322 (4)	Na35—Na36^ii^	3.8233 (18)
N16—C21	1.371 (4)	Na35—Na36	3.3934 (17)
N16—Na35	2.814 (3)	Na36—C32^ii^	2.913 (4)
C17—H17	0.9300	Na36—O33^ii^	2.549 (3)
C17—C18	1.395 (5)	Na36—O33^i^	2.290 (3)
C17—Na35	3.073 (4)	Na36—O34^ii^	2.567 (3)
C18—H18	0.9300	Na36—Na35^ii^	3.8233 (18)
C18—C19	1.364 (6)	Na36—Na36^iv^	3.554 (3)
C19—H19	0.9300	Na36—Na36^ii^	3.586 (3)
C19—C20	1.403 (5)	Na36—Na37^i^	3.4027 (16)
C20—C21	1.420 (4)	Na37—N1^iii^	2.769 (3)
C20—C22	1.420 (5)	Na37—C2^iii^	3.035 (4)
C21—C25	1.423 (4)	Na37—O14^iii^	2.334 (2)
C22—H22	0.9300	Na37—O30^ii^	2.288 (2)
C22—C23	1.366 (5)	Na37—Na36^iii^	3.4026 (16)
C23—H23	0.9300	Na37—Na36^ii^	3.8705 (18)
C23—C24	1.410 (5)		
			
C2—N1—C6	116.9 (3)	O29—Na35—Na36^ii^	107.87 (7)
C2—N1—Na37^i^	88.4 (2)	O29—Na35—Na37^v^	154.51 (7)
C6—N1—Na37^i^	111.3 (2)	O29—Na35—Na37^ii^	60.34 (6)
N1—C2—H2	118.0	O30^iii^—Na35—N16	112.27 (9)
N1—C2—C3	124.1 (3)	O30^iii^—Na35—C17	87.99 (9)
N1—C2—Na37^i^	65.77 (18)	O30^iii^—Na35—C27^iii^	22.17 (8)
C3—C2—H2	118.0	O30^iii^—Na35—O28^iii^	66.74 (8)
C3—C2—Na37^i^	122.0 (3)	O30^iii^—Na35—Na36	128.10 (7)
Na37^i^—C2—H2	82.7	O30^iii^—Na35—Na36^ii^	70.88 (6)
C2—C3—H3	120.4	O30^iii^—Na35—Na37^ii^	127.51 (7)
C4—C3—C2	119.1 (4)	O30^iii^—Na35—Na37^v^	30.11 (6)
C4—C3—H3	120.4	O34—Na35—O15^ii^	104.12 (10)
C3—C4—H4	120.0	O34—Na35—N16	156.15 (10)
C3—C4—C5	119.9 (3)	O34—Na35—C17	177.87 (10)
C5—C4—H4	120.0	O34—Na35—C27^iii^	84.64 (9)
C4—C5—C6	117.5 (3)	O34—Na35—O28^iii^	101.53 (9)
C4—C5—C7	123.3 (3)	O34—Na35—O29	83.84 (9)
C7—C5—C6	119.2 (3)	O34—Na35—O30^iii^	90.03 (9)
N1—C6—C5	122.5 (3)	O34—Na35—Na36^ii^	40.55 (7)
N1—C6—C10	118.4 (3)	O34—Na35—Na36	42.34 (6)
C5—C6—C10	119.1 (3)	O34—Na35—Na37^ii^	99.15 (7)
C5—C7—H7	119.4	O34—Na35—Na37^v^	85.38 (7)
C8—C7—C5	121.3 (3)	Na36—Na35—Na36^ii^	59.24 (4)
C8—C7—H7	119.4	Na36—Na35—Na37^v^	109.87 (4)
C7—C8—H8	120.2	Na36^ii^—Na35—Na37^ii^	83.36 (3)
C7—C8—C9	119.6 (3)	Na36—Na35—Na37^ii^	62.07 (3)
C9—C8—H8	120.2	Na36^ii^—Na35—Na37^v^	51.48 (3)
C8—C9—H9	119.0	Na37^v^—Na35—Na37^ii^	98.91 (4)
C10—C9—C8	122.0 (3)	O14—Na36—C32^ii^	117.07 (9)
C10—C9—H9	119.0	O14—Na36—Na35	128.39 (7)
C6—C10—C11	122.5 (3)	O14—Na36—Na35^ii^	63.15 (6)
C9—C10—C6	118.8 (3)	O14—Na36—Na36^iv^	110.54 (7)
C9—C10—C11	118.7 (3)	O14—Na36—Na36^ii^	96.09 (7)
C10—C11—C12	122.0 (3)	O14—Na36—Na37^i^	43.22 (5)
O13—C11—C10	120.5 (3)	O29—Na36—O14	128.38 (9)
O13—C11—C12	117.4 (3)	O29—Na36—C32^ii^	114.54 (9)
C11—C12—Na37	82.75 (17)	O29—Na36—O34^ii^	130.52 (9)
O14—C12—C11	116.5 (3)	O29—Na36—Na35	43.48 (5)
O14—C12—O15	128.5 (3)	O29—Na36—Na35^ii^	163.36 (7)
O14—C12—Na37	137.8 (2)	O29—Na36—Na36^iv^	96.27 (7)
O15—C12—C11	114.7 (3)	O29—Na36—Na36^ii^	109.56 (7)
O15—C12—Na37	46.18 (15)	O29—Na36—Na37^i^	129.57 (7)
C11—O13—Na37	106.3 (2)	C32^ii^—Na36—Na35	94.98 (7)
C12—O14—Na36	125.7 (2)	C32^ii^—Na36—Na35^ii^	55.28 (7)
C12—O14—Na37^i^	119.68 (19)	C32^ii^—Na36—Na36^iv^	59.35 (7)
Na37^i^—O14—Na36	86.79 (8)	C32^ii^—Na36—Na36^ii^	58.33 (7)
C12—O15—Na35^ii^	124.3 (2)	C32^ii^—Na36—Na37^i^	97.95 (7)
C12—O15—Na37	111.58 (19)	O33^ii^—Na36—O14	132.21 (9)
Na35^ii^—O15—Na37	119.72 (10)	O33^i^—Na36—O14	78.29 (9)
C17—N16—C21	117.8 (3)	O33^ii^—Na36—O29	96.64 (8)
C17—N16—Na35	88.3 (2)	O33^i^—Na36—O29	92.35 (8)
C21—N16—Na35	108.79 (19)	O33^ii^—Na36—C32^ii^	25.43 (8)
N16—C17—H17	118.0	O33^i^—Na36—C32^ii^	102.34 (10)
N16—C17—C18	123.9 (3)	O33^i^—Na36—O33^ii^	85.60 (10)
N16—C17—Na35	66.26 (18)	O33^i^—Na36—O34	156.71 (11)
C18—C17—H17	118.0	O33^i^—Na36—O34^ii^	116.40 (10)
C18—C17—Na35	119.8 (3)	O33^ii^—Na36—O34^ii^	50.81 (7)
Na35—C17—H17	84.3	O33^ii^—Na36—Na35^ii^	77.35 (6)
C17—C18—H18	120.5	O33^i^—Na36—Na35	135.58 (8)
C19—C18—C17	118.9 (3)	O33^i^—Na36—Na35^ii^	102.52 (7)
C19—C18—H18	120.5	O33^ii^—Na36—Na35	94.01 (7)
C18—C19—H19	120.1	O33^ii^—Na36—Na36^ii^	80.37 (7)
C18—C19—C20	119.9 (3)	O33^i^—Na36—Na36^iv^	45.64 (8)
C20—C19—H19	120.1	O33^i^—Na36—Na36^ii^	155.10 (9)
C19—C20—C21	117.6 (3)	O33^ii^—Na36—Na36^iv^	39.97 (6)
C19—C20—C22	123.2 (3)	O33^ii^—Na36—Na37^i^	98.05 (7)
C22—C20—C21	119.2 (3)	O33^i^—Na36—Na37^i^	41.57 (6)
N16—C21—C20	121.8 (3)	O34^ii^—Na36—O14	97.80 (8)
N16—C21—C25	118.8 (3)	O34—Na36—O14	90.80 (8)
C20—C21—C25	119.4 (3)	O34—Na36—O29	78.28 (8)
C20—C22—H22	119.6	O34^ii^—Na36—C32^ii^	25.40 (8)
C23—C22—C20	120.8 (3)	O34—Na36—C32^ii^	100.94 (10)
C23—C22—H22	119.6	O34—Na36—O33^ii^	116.40 (10)
C22—C23—H23	120.0	O34—Na36—O34^ii^	85.21 (10)
C22—C23—C24	120.0 (3)	O34^ii^—Na36—Na35^ii^	34.96 (5)
C24—C23—H23	120.0	O34^ii^—Na36—Na35	96.33 (6)
C23—C24—H24	119.3	O34—Na36—Na35^ii^	90.41 (7)
C25—C24—C23	121.3 (3)	O34—Na36—Na35	41.53 (6)
C25—C24—H24	119.3	O34—Na36—Na36^ii^	45.51 (7)
C21—C25—C26	121.9 (3)	O34^ii^—Na36—Na36^ii^	39.70 (6)
C24—C25—C21	119.3 (3)	O34^ii^—Na36—Na36^iv^	80.41 (7)
C24—C25—C26	118.8 (3)	O34—Na36—Na36^iv^	155.59 (9)
C25—C26—C27	121.5 (3)	O34^ii^—Na36—Na37^i^	94.98 (6)
O28—C26—C25	121.2 (3)	O34—Na36—Na37^i^	133.75 (8)
O28—C26—C27	117.2 (3)	Na35—Na36—Na35^ii^	120.76 (4)
C26—C27—Na35^i^	83.92 (17)	Na35—Na36—Na36^ii^	66.36 (4)
O29—C27—C26	116.2 (3)	Na35—Na36—Na36^iv^	120.68 (6)
O29—C27—O30	128.7 (3)	Na35—Na36—Na37^i^	167.03 (5)
O29—C27—Na35^i^	137.1 (2)	Na36^ii^—Na36—Na35^ii^	54.40 (4)
O30—C27—C26	114.9 (3)	Na36^iv^—Na36—Na35^ii^	89.01 (5)
O30—C27—Na35^i^	45.52 (15)	Na36^iv^—Na36—Na36^ii^	117.67 (7)
C26—O28—Na35^i^	105.9 (2)	Na37^i^—Na36—Na35^ii^	66.99 (4)
C27—O29—Na35	120.25 (19)	Na37^i^—Na36—Na36^ii^	120.36 (6)
C27—O29—Na36	126.7 (2)	Na37^i^—Na36—Na36^iv^	67.57 (4)
Na35—O29—Na36	87.60 (8)	N1^iii^—Na37—C2^iii^	25.79 (9)
C27—O30—Na35^i^	112.32 (19)	N1^iii^—Na37—C12	112.78 (9)
C27—O30—Na37^ii^	124.2 (2)	N1^iii^—Na37—Na35^ii^	118.43 (7)
Na37^ii^—O30—Na35^i^	118.63 (10)	N1^iii^—Na37—Na35^v^	75.29 (7)
H31A—C31—H31B	109.5	N1^iii^—Na37—Na36^iii^	119.97 (7)
H31A—C31—H31C	109.5	N1^iii^—Na37—Na36^ii^	153.15 (7)
H31B—C31—H31C	109.5	C2^iii^—Na37—C12	92.63 (10)
C32—C31—H31A	109.5	C2^iii^—Na37—Na35^ii^	93.41 (7)
C32—C31—H31B	109.5	C2^iii^—Na37—Na35^v^	84.17 (8)
C32—C31—H31C	109.5	C2^iii^—Na37—Na36^iii^	141.35 (9)
C31—C32—Na36^ii^	176.4 (3)	C2^iii^—Na37—Na36^ii^	138.44 (8)
O33—C32—C31	119.4 (3)	C12—Na37—Na35^ii^	50.62 (6)
O33—C32—Na36^ii^	60.79 (18)	C12—Na37—Na35^v^	149.24 (7)
O34—C32—C31	118.2 (3)	C12—Na37—Na36^iii^	125.90 (7)
O34—C32—O33	122.3 (3)	C12—Na37—Na36^ii^	80.15 (6)
O34—C32—Na36^ii^	61.64 (18)	O13—Na37—N1^iii^	80.90 (8)
C32—O33—Na36^iii^	130.7 (2)	O13—Na37—C2^iii^	76.26 (10)
C32—O33—Na36^ii^	93.8 (2)	O13—Na37—C12	47.68 (8)
C32—O33—Na37	127.0 (2)	O13—Na37—Na35^v^	155.72 (7)
Na36^iii^—O33—Na36^ii^	94.40 (10)	O13—Na37—Na35^ii^	96.49 (6)
Na37—O33—Na36^ii^	106.67 (10)	O13—Na37—Na36^ii^	122.07 (7)
Na37—O33—Na36^iii^	96.46 (10)	O13—Na37—Na36^iii^	130.05 (7)
C32—O34—Na35	131.2 (2)	O14^iii^—Na37—N1^iii^	73.69 (8)
C32—O34—Na36^ii^	93.0 (2)	O14^iii^—Na37—C2^iii^	99.20 (9)
C32—O34—Na36	127.8 (2)	O14^iii^—Na37—C12	149.10 (9)
Na35—O34—Na36^ii^	104.49 (10)	O14^iii^—Na37—O13	107.78 (9)
Na35—O34—Na36	96.13 (9)	O14^iii^—Na37—O15	170.06 (10)
Na36—O34—Na36^ii^	94.80 (10)	O14^iii^—Na37—Na35^v^	61.01 (6)
O15^ii^—Na35—N16	82.34 (9)	O14^iii^—Na37—Na35^ii^	154.67 (7)
O15^ii^—Na35—C17	76.91 (9)	O14^iii^—Na37—Na36^iii^	49.99 (6)
O15^ii^—Na35—C27^iii^	118.90 (9)	O14^iii^—Na37—Na36^ii^	107.83 (7)
O15^ii^—Na35—O28^iii^	149.41 (9)	O15—Na37—N1^iii^	112.82 (9)
O15^ii^—Na35—O29	91.21 (9)	O15—Na37—C2^iii^	88.02 (10)
O15^ii^—Na35—O30^iii^	96.73 (9)	O15—Na37—C12	22.25 (8)
O15^ii^—Na35—Na36^ii^	71.54 (7)	O15—Na37—O13	67.13 (8)
O15^ii^—Na35—Na36	81.21 (7)	O15—Na37—Na35^ii^	29.41 (6)
O15^ii^—Na35—Na37^ii^	30.87 (6)	O15—Na37—Na35^v^	127.04 (7)
O15^ii^—Na35—Na37^v^	69.18 (6)	O15—Na37—Na36^ii^	70.01 (6)
N16—Na35—C17	25.48 (9)	O15—Na37—Na36^iii^	126.03 (7)
N16—Na35—C27^iii^	112.70 (9)	O30^ii^—Na37—N1^iii^	82.82 (9)
N16—Na35—Na36	118.67 (7)	O30^ii^—Na37—C2^iii^	77.73 (10)
N16—Na35—Na36^ii^	153.86 (7)	O30^ii^—Na37—C12	118.21 (9)
N16—Na35—Na37^v^	118.11 (7)	O30^ii^—Na37—O13	149.27 (9)
N16—Na35—Na37^ii^	74.45 (6)	O30^ii^—Na37—O14^iii^	92.27 (9)
C17—Na35—Na36	139.79 (8)	O30^ii^—Na37—O15	95.96 (9)
C17—Na35—Na36^ii^	139.08 (8)	O30^ii^—Na37—Na35^v^	31.26 (6)
C17—Na35—Na37^v^	93.29 (7)	O30^ii^—Na37—Na35^ii^	68.97 (6)
C17—Na35—Na37^ii^	82.69 (7)	O30^ii^—Na37—Na36^ii^	70.37 (7)
C27^iii^—Na35—C17	93.22 (9)	O30^ii^—Na37—Na36^iii^	80.69 (6)
C27^iii^—Na35—Na36	126.95 (7)	O33—Na37—N1^iii^	158.05 (10)
C27^iii^—Na35—Na36^ii^	80.24 (7)	O33—Na37—C2^iii^	176.10 (11)
C27^iii^—Na35—Na37^ii^	149.63 (7)	O33—Na37—C12	83.95 (9)
C27^iii^—Na35—Na37^v^	51.11 (6)	O33—Na37—O13	102.63 (9)
O28^iii^—Na35—N16	80.90 (8)	O33—Na37—O14^iii^	84.70 (9)
O28^iii^—Na35—C17	76.96 (9)	O33—Na37—O15	88.11 (9)
O28^iii^—Na35—C27^iii^	47.57 (7)	O33—Na37—O30^ii^	102.21 (10)
O28^iii^—Na35—Na36^ii^	121.72 (6)	O33—Na37—Na35^ii^	82.97 (7)
O28^iii^—Na35—Na36	129.36 (7)	O33—Na37—Na35^v^	97.80 (7)
O28^iii^—Na35—Na37^ii^	154.89 (7)	O33—Na37—Na36^ii^	39.11 (7)
O28^iii^—Na35—Na37^v^	96.80 (6)	O33—Na37—Na36^iii^	41.96 (6)
O29—Na35—N16	72.98 (8)	Na35^v^—Na37—Na35^ii^	98.91 (4)
O29—Na35—C17	98.04 (9)	Na36^iii^—Na37—Na35^ii^	108.19 (4)
O29—Na35—C27^iii^	149.60 (9)	Na36^ii^—Na37—Na35^v^	82.16 (3)
O29—Na35—O28^iii^	107.95 (8)	Na36^iii^—Na37—Na35^v^	61.53 (3)
O29—Na35—O30^iii^	170.97 (10)	Na36^ii^—Na37—Na35^ii^	50.77 (3)
O29—Na35—Na36	48.92 (6)	Na36^iii^—Na37—Na36^ii^	58.08 (4)
			
N1—C2—C3—C4	0.9 (7)	C27^iii^—Na35—Na36—Na35^ii^	−46.01 (9)
N1—C6—C10—C9	−178.4 (3)	C27^iii^—Na35—Na36—Na36^ii^	−46.01 (9)
N1—C6—C10—C11	2.9 (5)	C27^iii^—Na35—Na36—Na36^iv^	−155.26 (9)
C2—N1—C6—C5	−1.1 (5)	C27^iii^—Na35—Na36—Na37^i^	77.9 (3)
C2—N1—C6—C10	177.9 (3)	O28—C26—C27—O29	−118.7 (3)
C2—C3—C4—C5	−0.3 (7)	O28—C26—C27—O30	55.8 (4)
C3—C4—C5—C6	−0.9 (6)	O28—C26—C27—Na35^i^	21.5 (3)
C3—C4—C5—C7	−179.3 (4)	O28^iii^—Na35—Na36—O14	−29.13 (13)
C4—C5—C6—N1	1.7 (5)	O28^iii^—Na35—Na36—O29	79.27 (11)
C4—C5—C6—C10	−177.3 (3)	O28^iii^—Na35—Na36—C32^ii^	−159.81 (10)
C4—C5—C7—C8	177.2 (4)	O28^iii^—Na35—Na36—O33^ii^	174.69 (9)
C5—C6—C10—C9	0.6 (5)	O28^iii^—Na35—Na36—O33^i^	86.89 (14)
C5—C6—C10—C11	−178.1 (3)	O28^iii^—Na35—Na36—O34	−58.92 (13)
C5—C7—C8—C9	−0.5 (5)	O28^iii^—Na35—Na36—O34^ii^	−134.32 (9)
C6—N1—C2—C3	−0.2 (6)	O28^iii^—Na35—Na36—Na35^ii^	−107.65 (9)
C6—N1—C2—Na37^i^	113.2 (3)	O28^iii^—Na35—Na36—Na36^ii^	−107.65 (9)
C6—C5—C7—C8	−1.1 (5)	O28^iii^—Na35—Na36—Na36^iv^	143.10 (9)
C6—C10—C11—C12	28.7 (5)	O28^iii^—Na35—Na36—Na37^i^	16.3 (3)
C6—C10—C11—O13	−155.2 (3)	O29—C27—O30—Na35^i^	121.9 (3)
C7—C5—C6—N1	−179.9 (3)	O29—C27—O30—Na37^ii^	−32.8 (4)
C7—C5—C6—C10	1.1 (5)	O29—Na35—Na36—O14	−108.40 (12)
C7—C8—C9—C10	2.3 (5)	O29—Na35—Na36—C32^ii^	120.92 (11)
C8—C9—C10—C6	−2.4 (5)	O29—Na35—Na36—O33^i^	7.62 (14)
C8—C9—C10—C11	176.4 (3)	O29—Na35—Na36—O33^ii^	95.43 (10)
C9—C10—C11—C12	−150.0 (3)	O29—Na35—Na36—O34^ii^	146.41 (10)
C9—C10—C11—O13	26.1 (4)	O29—Na35—Na36—O34	−138.19 (14)
C10—C11—C12—O14	58.0 (4)	O29—Na35—Na36—Na35^ii^	173.08 (10)
C10—C11—C12—O15	−127.3 (3)	O29—Na35—Na36—Na36^ii^	173.08 (10)
C10—C11—C12—Na37	−161.5 (3)	O29—Na35—Na36—Na36^iv^	63.83 (10)
C10—C11—O13—Na37	157.1 (2)	O29—Na35—Na36—Na37^i^	−63.0 (2)
C11—C12—O14—Na36	148.1 (2)	O30—C27—O29—Na35	84.3 (4)
C11—C12—O14—Na37^i^	−102.7 (3)	O30—C27—O29—Na36	−27.7 (4)
C11—C12—O15—Na35^ii^	153.0 (2)	O30^iii^—Na35—Na36—O14	60.53 (13)
C11—C12—O15—Na37	−50.6 (3)	O30^iii^—Na35—Na36—O29	168.92 (13)
C11—C12—Na37—N1^iii^	40.10 (18)	O30^iii^—Na35—Na36—C32^ii^	−70.16 (11)
C11—C12—Na37—C2^iii^	56.71 (18)	O30^iii^—Na35—Na36—O33^i^	176.54 (14)
C11—C12—Na37—O13	−11.98 (15)	O30^iii^—Na35—Na36—O33^ii^	−95.65 (11)
C11—C12—Na37—O14^iii^	−56.1 (2)	O30^iii^—Na35—Na36—O34^ii^	−44.66 (11)
C11—C12—Na37—O15	134.9 (3)	O30^iii^—Na35—Na36—O34	30.74 (13)
C11—C12—Na37—O30^ii^	134.19 (17)	O30^iii^—Na35—Na36—Na35^ii^	−17.99 (10)
C11—C12—Na37—O33	−125.17 (18)	O30^iii^—Na35—Na36—Na36^iv^	−127.25 (10)
C11—C12—Na37—Na35^ii^	148.96 (19)	O30^iii^—Na35—Na36—Na36^ii^	−17.99 (10)
C11—C12—Na37—Na35^v^	139.73 (16)	O30^iii^—Na35—Na36—Na37^i^	105.9 (2)
C11—C12—Na37—Na36^iii^	−126.51 (16)	C31—C32—O33—Na36^ii^	175.8 (3)
C11—C12—Na37—Na36^ii^	−164.50 (17)	C31—C32—O33—Na36^iii^	−85.1 (4)
C11—O13—Na37—N1^iii^	−117.1 (2)	C31—C32—O33—Na37	61.6 (4)
C11—O13—Na37—C2^iii^	−91.2 (2)	C31—C32—O34—Na35	71.9 (4)
C11—O13—Na37—C12	15.48 (19)	C31—C32—O34—Na36	−77.5 (4)
C11—O13—Na37—O14^iii^	173.4 (2)	C31—C32—O34—Na36^ii^	−175.9 (3)
C11—O13—Na37—O15	2.5 (2)	C32—O33—Na37—N1^iii^	−114.8 (3)
C11—O13—Na37—O30^ii^	−58.2 (3)	C32—O33—Na37—C12	26.4 (3)
C11—O13—Na37—O33	85.0 (2)	C32—O33—Na37—O13	−17.8 (3)
C11—O13—Na37—Na35^ii^	0.8 (2)	C32—O33—Na37—O14^iii^	−124.8 (3)
C11—O13—Na37—Na35^v^	−128.4 (2)	C32—O33—Na37—O15	48.3 (3)
C11—O13—Na37—Na36^ii^	47.9 (2)	C32—O33—Na37—O30^ii^	144.0 (3)
C11—O13—Na37—Na36^iii^	121.2 (2)	C32—O33—Na37—Na35^ii^	77.3 (3)
C12—C11—O13—Na37	−26.6 (3)	C32—O33—Na37—Na35^v^	175.4 (3)
C12—O14—Na36—O29	−125.1 (2)	C32—O33—Na37—Na36^ii^	108.2 (3)
C12—O14—Na36—C32^ii^	53.2 (3)	C32—O33—Na37—Na36^iii^	−155.2 (3)
C12—O14—Na36—O33^i^	151.2 (2)	C32—O34—Na35—O15^ii^	144.5 (3)
C12—O14—Na36—O33^ii^	78.3 (3)	C32—O34—Na35—N16	−112.2 (3)
C12—O14—Na36—O34^ii^	35.8 (2)	C32—O34—Na35—C27^iii^	26.1 (3)
C12—O14—Na36—O34	−49.5 (2)	C32—O34—Na35—O28^iii^	−18.6 (3)
C12—O14—Na36—Na35^ii^	40.6 (2)	C32—O34—Na35—O29	−125.8 (3)
C12—O14—Na36—Na35	−68.7 (3)	C32—O34—Na35—O30^iii^	47.6 (3)
C12—O14—Na36—Na36^ii^	−4.2 (2)	C32—O34—Na35—Na36^ii^	107.3 (3)
C12—O14—Na36—Na36^iv^	118.4 (2)	C32—O34—Na35—Na36	−156.1 (3)
C12—O14—Na36—Na37^i^	124.8 (3)	C32—O34—Na35—Na37^ii^	175.7 (3)
C12—O15—Na37—N1^iii^	94.6 (2)	C32—O34—Na35—Na37^v^	77.4 (3)
C12—O15—Na37—C2^iii^	101.9 (2)	C32—O34—Na36—O14	0.2 (3)
C12—O15—Na37—O13	26.0 (2)	C32—O34—Na36—O29	129.4 (3)
C12—O15—Na37—O30^ii^	179.3 (2)	C32—O34—Na36—C32^ii^	−117.5 (3)
C12—O15—Na37—O33	−78.6 (2)	C32—O34—Na36—O33^ii^	−139.0 (3)
C12—O15—Na37—Na35^ii^	−157.6 (3)	C32—O34—Na36—O33^i^	61.5 (4)
C12—O15—Na37—Na35^v^	−176.94 (18)	C32—O34—Na36—O34^ii^	−97.5 (3)
C12—O15—Na37—Na36^iii^	−97.9 (2)	C32—O34—Na36—Na35	157.3 (3)
C12—O15—Na37—Na36^ii^	−114.1 (2)	C32—O34—Na36—Na35^ii^	−62.9 (3)
O13—C11—C12—O14	−118.2 (3)	C32—O34—Na36—Na36^iv^	−151.4 (3)
O13—C11—C12—O15	56.5 (4)	C32—O34—Na36—Na36^ii^	−97.5 (3)
O13—C11—C12—Na37	22.3 (3)	C32—O34—Na36—Na37^i^	−5.2 (3)
O14—C12—O15—Na35^ii^	−33.0 (4)	O33—C32—O34—Na35	−109.0 (3)
O14—C12—O15—Na37	123.3 (3)	O33—C32—O34—Na36^ii^	3.2 (3)
O14—C12—Na37—N1^iii^	162.1 (3)	O33—C32—O34—Na36	101.6 (3)
O14—C12—Na37—C2^iii^	178.7 (3)	O34—C32—O33—Na36^iii^	95.9 (4)
O14—C12—Na37—O13	110.0 (3)	O34—C32—O33—Na36^ii^	−3.3 (3)
O14—C12—Na37—O14^iii^	65.9 (4)	O34—C32—O33—Na37	−117.5 (3)
O14—C12—Na37—O15	−103.0 (4)	O34—Na35—Na36—O14	29.79 (12)
O14—C12—Na37—O30^ii^	−103.8 (3)	O34—Na35—Na36—O29	138.19 (14)
O14—C12—Na37—O33	−3.1 (3)	O34—Na35—Na36—C32^ii^	−100.89 (12)
O14—C12—Na37—Na35^v^	−98.2 (3)	O34—Na35—Na36—O33^i^	145.81 (17)
O14—C12—Na37—Na35^ii^	−89.0 (3)	O34—Na35—Na36—O33^ii^	−126.39 (12)
O14—C12—Na37—Na36^iii^	−4.5 (4)	O34—Na35—Na36—O34^ii^	−75.40 (13)
O14—C12—Na37—Na36^ii^	−42.5 (3)	O34—Na35—Na36—Na35^ii^	−48.73 (10)
O15—C12—O14—Na36	−25.8 (4)	O34—Na35—Na36—Na36^ii^	−48.73 (10)
O15—C12—O14—Na37^i^	83.5 (4)	O34—Na35—Na36—Na36^iv^	−157.98 (13)
O15—C12—Na37—N1^iii^	−94.8 (2)	O34—Na35—Na36—Na37^i^	75.2 (2)
O15—C12—Na37—C2^iii^	−78.2 (2)	Na35^ii^—O15—Na37—N1^iii^	−107.78 (12)
O15—C12—Na37—O13	−146.9 (2)	Na35^ii^—O15—Na37—C2^iii^	−100.52 (13)
O15—C12—Na37—O14^iii^	168.9 (2)	Na35^ii^—O15—Na37—C12	157.6 (3)
O15—C12—Na37—O30^ii^	−0.8 (2)	Na35^ii^—O15—Na37—O13	−176.45 (14)
O15—C12—Na37—O33	99.9 (2)	Na35^ii^—O15—Na37—O30^ii^	−23.08 (13)
O15—C12—Na37—Na35^v^	4.8 (3)	Na35^ii^—O15—Na37—O33	79.00 (13)
O15—C12—Na37—Na35^ii^	14.02 (18)	Na35^ii^—O15—Na37—Na35^v^	−19.35 (15)
O15—C12—Na37—Na36^iii^	98.5 (2)	Na35^ii^—O15—Na37—Na36^iii^	59.73 (14)
O15—C12—Na37—Na36^ii^	60.6 (2)	Na35^ii^—O15—Na37—Na36^ii^	43.51 (10)
O15^ii^—Na35—Na36—O14	152.22 (10)	Na35—N16—C17—C18	−110.9 (4)
O15^ii^—Na35—Na36—O29	−99.39 (10)	Na35—N16—C21—C20	96.9 (3)
O15^ii^—Na35—Na36—C32^ii^	21.53 (9)	Na35—N16—C21—C25	−83.1 (3)
O15^ii^—Na35—Na36—O33^ii^	−3.96 (9)	Na35—C17—C18—C19	−79.3 (4)
O15^ii^—Na35—Na36—O33^i^	−91.77 (14)	Na35^i^—C27—O29—Na35	147.06 (19)
O15^ii^—Na35—Na36—O34	122.42 (13)	Na35^i^—C27—O29—Na36	35.1 (4)
O15^ii^—Na35—Na36—O34^ii^	47.02 (9)	Na35^i^—C27—O30—Na37^ii^	−154.8 (3)
O15^ii^—Na35—Na36—Na35^ii^	73.70 (7)	Na35—O29—Na36—O14	108.43 (10)
O15^ii^—Na35—Na36—Na36^ii^	73.70 (7)	Na35—O29—Na36—C32^ii^	−69.96 (10)
O15^ii^—Na35—Na36—Na36^iv^	−35.56 (9)	Na35—O29—Na36—O33^i^	−174.67 (10)
O15^ii^—Na35—Na36—Na37^i^	−162.4 (2)	Na35—O29—Na36—O33^ii^	−88.83 (9)
N16—C17—C18—C19	0.7 (6)	Na35—O29—Na36—O34	26.83 (9)
N16—C17—Na35—O15^ii^	99.8 (2)	Na35—O29—Na36—O34^ii^	−46.33 (13)
N16—C17—Na35—C27^iii^	−141.3 (2)	Na35—O29—Na36—Na35^ii^	−21.2 (3)
N16—C17—Na35—O28^iii^	−96.2 (2)	Na35—O29—Na36—Na36^ii^	−6.72 (9)
N16—C17—Na35—O29	10.4 (2)	Na35—O29—Na36—Na36^iv^	−129.06 (7)
N16—C17—Na35—O30^iii^	−162.8 (2)	Na35—O29—Na36—Na37^i^	164.97 (7)
N16—C17—Na35—Na36	41.0 (2)	Na35—O34—Na36—O14	−157.08 (9)
N16—C17—Na35—Na36^ii^	139.93 (17)	Na35—O34—Na36—O29	−27.94 (9)
N16—C17—Na35—Na37^v^	167.53 (19)	Na35—O34—Na36—C32^ii^	85.13 (10)
N16—C17—Na35—Na37^ii^	68.96 (19)	Na35—O34—Na36—O33^i^	−95.8 (3)
N16—C21—C25—C24	−179.4 (3)	Na35—O34—Na36—O33^ii^	63.71 (12)
N16—C21—C25—C26	1.9 (4)	Na35—O34—Na36—O34^ii^	105.16 (11)
N16—Na35—Na36—O14	−131.57 (11)	Na35—O34—Na36—Na35^ii^	139.77 (8)
N16—Na35—Na36—O29	−23.17 (10)	Na35—O34—Na36—Na36^iv^	51.3 (3)
N16—Na35—Na36—C32^ii^	97.75 (10)	Na35—O34—Na36—Na36^ii^	105.16 (11)
N16—Na35—Na36—O33^i^	−15.55 (15)	Na35—O34—Na36—Na37^i^	−162.53 (7)
N16—Na35—Na36—O33^ii^	72.25 (10)	Na36—O29—Na35—O15^ii^	77.22 (9)
N16—Na35—Na36—O34^ii^	123.24 (9)	Na36—O29—Na35—N16	158.84 (9)
N16—Na35—Na36—O34	−161.36 (13)	Na36—O29—Na35—C17	154.17 (9)
N16—Na35—Na36—Na35^ii^	149.91 (8)	Na36—O29—Na35—C27^iii^	−95.16 (17)
N16—Na35—Na36—Na36^ii^	149.91 (8)	Na36—O29—Na35—O28^iii^	−127.01 (8)
N16—Na35—Na36—Na36^iv^	40.66 (10)	Na36—O29—Na35—O34	−26.85 (9)
N16—Na35—Na36—Na37^i^	−86.2 (2)	Na36—O29—Na35—Na36^ii^	6.24 (9)
C17—N16—C21—C20	−1.3 (5)	Na36—O29—Na35—Na37^v^	38.65 (19)
C17—N16—C21—C25	178.6 (3)	Na36—O29—Na35—Na37^ii^	77.37 (6)
C17—N16—Na35—O15^ii^	−75.6 (2)	Na36^ii^—C32—O33—Na36^iii^	99.1 (3)
C17—N16—Na35—C27^iii^	42.6 (2)	Na36^ii^—C32—O33—Na37	−114.2 (2)
C17—N16—Na35—O28^iii^	78.7 (2)	Na36^ii^—C32—O34—Na35	−112.2 (3)
C17—N16—Na35—O29	−169.2 (2)	Na36^ii^—C32—O34—Na36	98.4 (2)
C17—N16—Na35—O30^iii^	18.6 (2)	Na36^ii^—O33—Na37—N1^iii^	137.0 (2)
C17—N16—Na35—O34	176.7 (3)	Na36^iii^—O33—Na37—N1^iii^	40.5 (3)
C17—N16—Na35—Na36	−151.13 (18)	Na36^iii^—O33—Na37—C12	−178.38 (11)
C17—N16—Na35—Na36^ii^	−73.2 (3)	Na36^ii^—O33—Na37—C12	−81.81 (10)
C17—N16—Na35—Na37^ii^	−106.1 (2)	Na36^iii^—O33—Na37—O13	137.47 (9)
C17—N16—Na35—Na37^v^	−14.1 (2)	Na36^ii^—O33—Na37—O13	−125.95 (9)
C17—C18—C19—C20	1.1 (6)	Na36^ii^—O33—Na37—O14^iii^	126.98 (10)
C17—Na35—Na36—O14	−150.33 (13)	Na36^iii^—O33—Na37—O14^iii^	30.41 (10)
C17—Na35—Na36—O29	−41.94 (14)	Na36^ii^—O33—Na37—O15	−59.89 (10)
C17—Na35—Na36—C32^ii^	78.98 (14)	Na36^iii^—O33—Na37—O15	−156.47 (10)
C17—Na35—Na36—O33^i^	−34.32 (18)	Na36^ii^—O33—Na37—O30^ii^	35.80 (11)
C17—Na35—Na36—O33^ii^	53.49 (14)	Na36^iii^—O33—Na37—O30^ii^	−60.78 (11)
C17—Na35—Na36—O34	179.87 (16)	Na36^ii^—O33—Na37—Na35^v^	67.25 (9)
C17—Na35—Na36—O34^ii^	104.48 (13)	Na36^iii^—O33—Na37—Na35^ii^	−127.41 (9)
C17—Na35—Na36—Na35^ii^	131.15 (12)	Na36^iii^—O33—Na37—Na35^v^	−29.33 (10)
C17—Na35—Na36—Na36^iv^	21.89 (15)	Na36^ii^—O33—Na37—Na35^ii^	−30.84 (8)
C17—Na35—Na36—Na36^ii^	131.15 (12)	Na36^ii^—O33—Na37—Na36^iii^	96.57 (12)
C17—Na35—Na36—Na37^i^	−104.9 (2)	Na36^iii^—O33—Na37—Na36^ii^	−96.57 (12)
C18—C17—Na35—O15^ii^	−143.4 (3)	Na36—O34—Na35—O15^ii^	−59.34 (11)
C18—C17—Na35—N16	116.8 (4)	Na36^ii^—O34—Na35—O15^ii^	37.24 (11)
C18—C17—Na35—C27^iii^	−24.5 (3)	Na36^ii^—O34—Na35—N16	140.5 (2)
C18—C17—Na35—O28^iii^	20.6 (3)	Na36—O34—Na35—N16	43.9 (3)
C18—C17—Na35—O29	127.2 (3)	Na36^ii^—O34—Na35—C27^iii^	−81.24 (9)
C18—C17—Na35—O30^iii^	−46.0 (3)	Na36—O34—Na35—C27^iii^	−177.82 (10)
C18—C17—Na35—Na36^ii^	−103.3 (3)	Na36—O34—Na35—O28^iii^	137.48 (9)
C18—C17—Na35—Na36	157.8 (2)	Na36^ii^—O34—Na35—O28^iii^	−125.94 (9)
C18—C17—Na35—Na37^ii^	−174.2 (3)	Na36^ii^—O34—Na35—O29	126.94 (10)
C18—C17—Na35—Na37^v^	−75.7 (3)	Na36—O34—Na35—O29	30.36 (10)
C18—C19—C20—C21	−2.8 (6)	Na36—O34—Na35—O30^iii^	−156.29 (10)
C18—C19—C20—C22	178.7 (4)	Na36^ii^—O34—Na35—O30^iii^	−59.70 (10)
C19—C20—C21—N16	3.0 (5)	Na36—O34—Na35—Na36^ii^	−96.58 (11)
C19—C20—C21—C25	−177.0 (3)	Na36^ii^—O34—Na35—Na36	96.58 (11)
C19—C20—C22—C23	176.9 (4)	Na36^ii^—O34—Na35—Na37^v^	−29.93 (7)
C20—C21—C25—C24	0.5 (5)	Na36—O34—Na35—Na37^v^	−126.51 (8)
C20—C21—C25—C26	−178.2 (3)	Na36—O34—Na35—Na37^ii^	−28.20 (9)
C20—C22—C23—C24	−0.6 (5)	Na36^ii^—O34—Na35—Na37^ii^	68.38 (8)
C21—N16—C17—C18	−0.6 (5)	Na36^ii^—O34—Na36—O14	97.76 (8)
C21—N16—C17—Na35	110.4 (3)	Na36^ii^—O34—Na36—O29	−133.10 (9)
C21—N16—Na35—O15^ii^	165.5 (2)	Na36^ii^—O34—Na36—C32^ii^	−20.03 (9)
C21—N16—Na35—C17	−118.9 (3)	Na36^ii^—O34—Na36—O33^i^	159.0 (2)
C21—N16—Na35—C27^iii^	−76.3 (2)	Na36^ii^—O34—Na36—O33^ii^	−41.45 (10)
C21—N16—Na35—O28^iii^	−40.1 (2)	Na36^ii^—O34—Na36—O34^ii^	0.0
C21—N16—Na35—O29	71.9 (2)	Na36^ii^—O34—Na36—Na35^ii^	34.61 (6)
C21—N16—Na35—O30^iii^	−100.3 (2)	Na36^ii^—O34—Na36—Na35	−105.16 (11)
C21—N16—Na35—O34	57.8 (3)	Na36^ii^—O34—Na36—Na36^iv^	−53.9 (2)
C21—N16—Na35—Na36	90.0 (2)	Na36^ii^—O34—Na36—Na37^i^	92.31 (11)
C21—N16—Na35—Na36^ii^	167.92 (17)	Na36^ii^—Na35—Na36—O14	78.52 (9)
C21—N16—Na35—Na37^v^	−133.04 (18)	Na36^ii^—Na35—Na36—O29	−173.08 (10)
C21—N16—Na35—Na37^ii^	135.0 (2)	Na36^ii^—Na35—Na36—C32^ii^	−52.16 (7)
C21—C20—C22—C23	−1.6 (5)	Na36^ii^—Na35—Na36—O33^ii^	−77.66 (7)
C21—C25—C26—C27	31.5 (4)	Na36^ii^—Na35—Na36—O33^i^	−165.47 (14)
C21—C25—C26—O28	−153.1 (3)	Na36^ii^—Na35—Na36—O34^ii^	−26.67 (5)
C22—C20—C21—N16	−178.4 (3)	Na36^ii^—Na35—Na36—O34	48.73 (10)
C22—C20—C21—C25	1.6 (5)	Na36^ii^—Na35—Na36—Na35^ii^	0.0
C22—C23—C24—C25	2.9 (5)	Na36^ii^—Na35—Na36—Na36^iv^	−109.25 (8)
C23—C24—C25—C21	−2.8 (5)	Na36^ii^—Na35—Na36—Na37^i^	123.9 (2)
C23—C24—C25—C26	176.0 (3)	Na37^i^—N1—C2—C3	−113.3 (4)
C24—C25—C26—C27	−147.2 (3)	Na37^i^—N1—C6—C5	98.4 (3)
C24—C25—C26—O28	28.1 (4)	Na37^i^—N1—C6—C10	−82.6 (3)
C25—C26—C27—O29	56.8 (4)	Na37^i^—C2—C3—C4	−79.8 (5)
C25—C26—C27—O30	−128.7 (3)	Na37—C12—O14—Na36	38.1 (4)
C25—C26—C27—Na35^i^	−163.0 (3)	Na37—C12—O14—Na37^i^	147.35 (19)
C25—C26—O28—Na35^i^	159.2 (2)	Na37—C12—O15—Na35^ii^	−156.4 (3)
C26—C27—O29—Na35	−102.1 (3)	Na37^i^—O14—Na36—O29	110.11 (10)
C26—C27—O29—Na36	145.9 (2)	Na37^i^—O14—Na36—C32^ii^	−71.52 (11)
C26—C27—O30—Na35^i^	−51.8 (3)	Na37^i^—O14—Na36—O33^i^	26.48 (9)
C26—C27—O30—Na37^ii^	153.47 (19)	Na37^i^—O14—Na36—O33^ii^	−46.44 (14)
C27—C26—O28—Na35^i^	−25.2 (3)	Na37^i^—O14—Na36—O34	−174.25 (9)
C27—O29—Na35—O15^ii^	−54.6 (2)	Na37^i^—O14—Na36—O34^ii^	−88.98 (9)
C27—O29—Na35—N16	27.0 (2)	Na37^i^—O14—Na36—Na35^ii^	−84.20 (7)
C27—O29—Na35—C17	22.3 (2)	Na37^i^—O14—Na36—Na35	166.51 (7)
C27—O29—Na35—C27^iii^	133.0 (3)	Na37^i^—O14—Na36—Na36^iv^	−6.35 (9)
C27—O29—Na35—O28^iii^	101.1 (2)	Na37^i^—O14—Na36—Na36^ii^	−128.95 (7)
C27—O29—Na35—O34	−158.7 (2)	Na37^v^—Na35—Na36—O14	88.21 (9)
C27—O29—Na35—Na36^ii^	−125.6 (2)	Na37^ii^—Na35—Na36—O14	177.91 (9)
C27—O29—Na35—Na36	−131.9 (3)	Na37^ii^—Na35—Na36—O29	−73.69 (8)
C27—O29—Na35—Na37^v^	−93.2 (3)	Na37^v^—Na35—Na36—O29	−163.40 (9)
C27—O29—Na35—Na37^ii^	−54.5 (2)	Na37^v^—Na35—Na36—C32^ii^	−42.48 (8)
C27—O29—Na36—O14	−124.9 (2)	Na37^ii^—Na35—Na36—C32^ii^	47.23 (7)
C27—O29—Na36—C32^ii^	56.7 (3)	Na37^v^—Na35—Na36—O33^ii^	−67.97 (7)
C27—O29—Na36—O33^i^	−48.0 (2)	Na37^ii^—Na35—Na36—O33^ii^	21.73 (6)
C27—O29—Na36—O33^ii^	37.8 (2)	Na37^ii^—Na35—Na36—O33^i^	−66.07 (12)
C27—O29—Na36—O34	153.5 (2)	Na37^v^—Na35—Na36—O33^i^	−155.78 (12)
C27—O29—Na36—O34^ii^	80.3 (3)	Na37^ii^—Na35—Na36—O34^ii^	72.72 (6)
C27—O29—Na36—Na35	126.7 (3)	Na37^v^—Na35—Na36—O34^ii^	−16.98 (7)
C27—O29—Na36—Na35^ii^	105.5 (3)	Na37^ii^—Na35—Na36—O34	148.12 (11)
C27—O29—Na36—Na36^iv^	−2.4 (2)	Na37^v^—Na35—Na36—O34	58.42 (11)
C27—O29—Na36—Na36^ii^	120.0 (2)	Na37^v^—Na35—Na36—Na35^ii^	9.69 (5)
C27—O29—Na36—Na37^i^	−68.4 (3)	Na37^ii^—Na35—Na36—Na35^ii^	99.39 (5)
C27^iii^—Na35—Na36—O14	32.51 (13)	Na37^ii^—Na35—Na36—Na36^iv^	−9.86 (6)
C27^iii^—Na35—Na36—O29	140.91 (12)	Na37^v^—Na35—Na36—Na36^iv^	−99.57 (7)
C27^iii^—Na35—Na36—C32^ii^	−98.17 (11)	Na37^ii^—Na35—Na36—Na36^ii^	99.39 (5)
C27^iii^—Na35—Na36—O33^i^	148.52 (14)	Na37^v^—Na35—Na36—Na36^ii^	9.69 (5)
C27^iii^—Na35—Na36—O33^ii^	−123.67 (10)	Na37^v^—Na35—Na36—Na37^i^	133.6 (2)
C27^iii^—Na35—Na36—O34^ii^	−72.68 (10)	Na37^ii^—Na35—Na36—Na37^i^	−136.7 (2)
C27^iii^—Na35—Na36—O34	2.72 (13)		

Symmetry codes: (i) *x*−1, *y*, *z*; (ii) −*x*+1, −*y*+1, −*z*; (iii) *x*+1, *y*, *z*; (iv) −*x*, −*y*+1, −*z*; (v) −*x*+2, −*y*+1, −*z*.
