# Supplementary figures and images for: Crystal structure of poly[μ-acetato-bis­[μ-2-oxo-2-(quinolin-8-yl)ethano­ato]tris­odium]
Source: Acta Crystallogr Sect E Struct Rep Online. 2014 Oct 31;70(Pt 11):m385–6. doi: 10.1107/S1600536814023423 (PMC4257345; doi:10.1107/S1600536814023423)

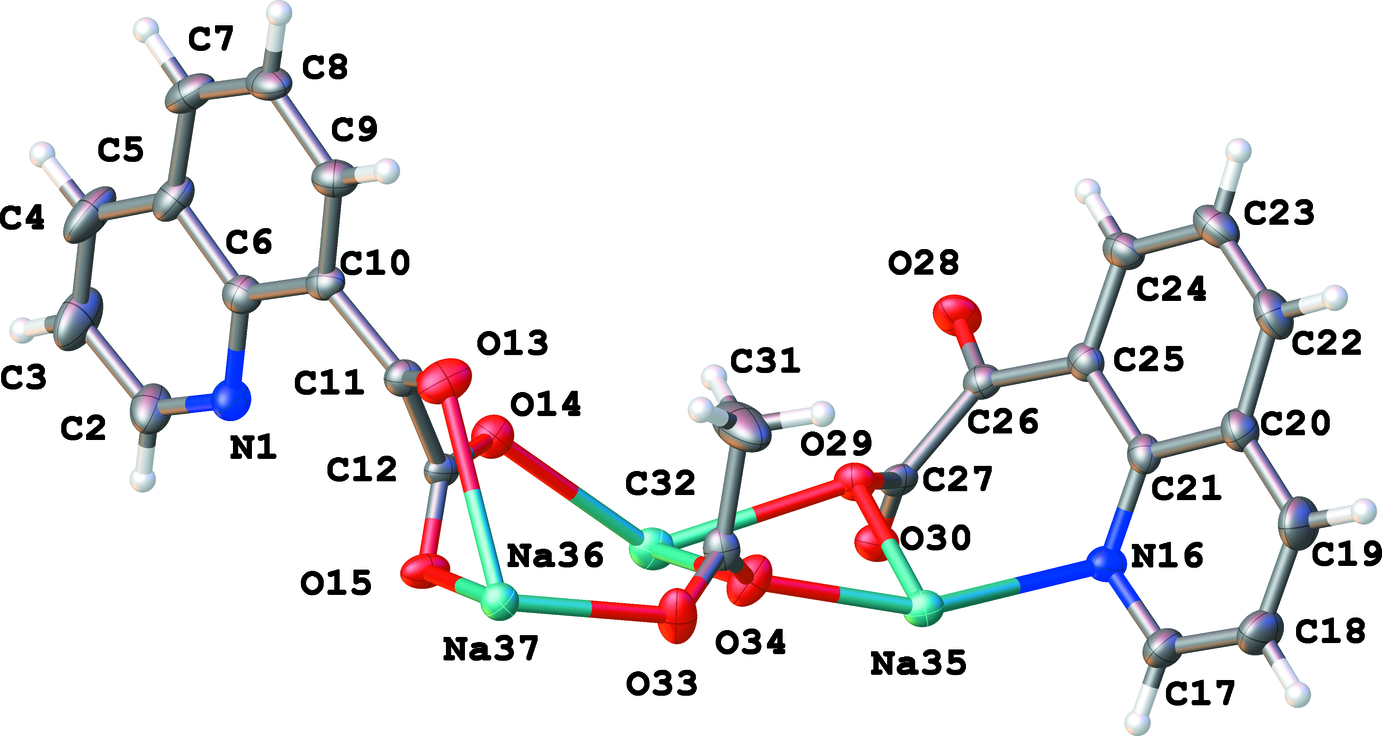

Supplement: Supplementary file 3 [file e-70-0m385-fig1.tif]

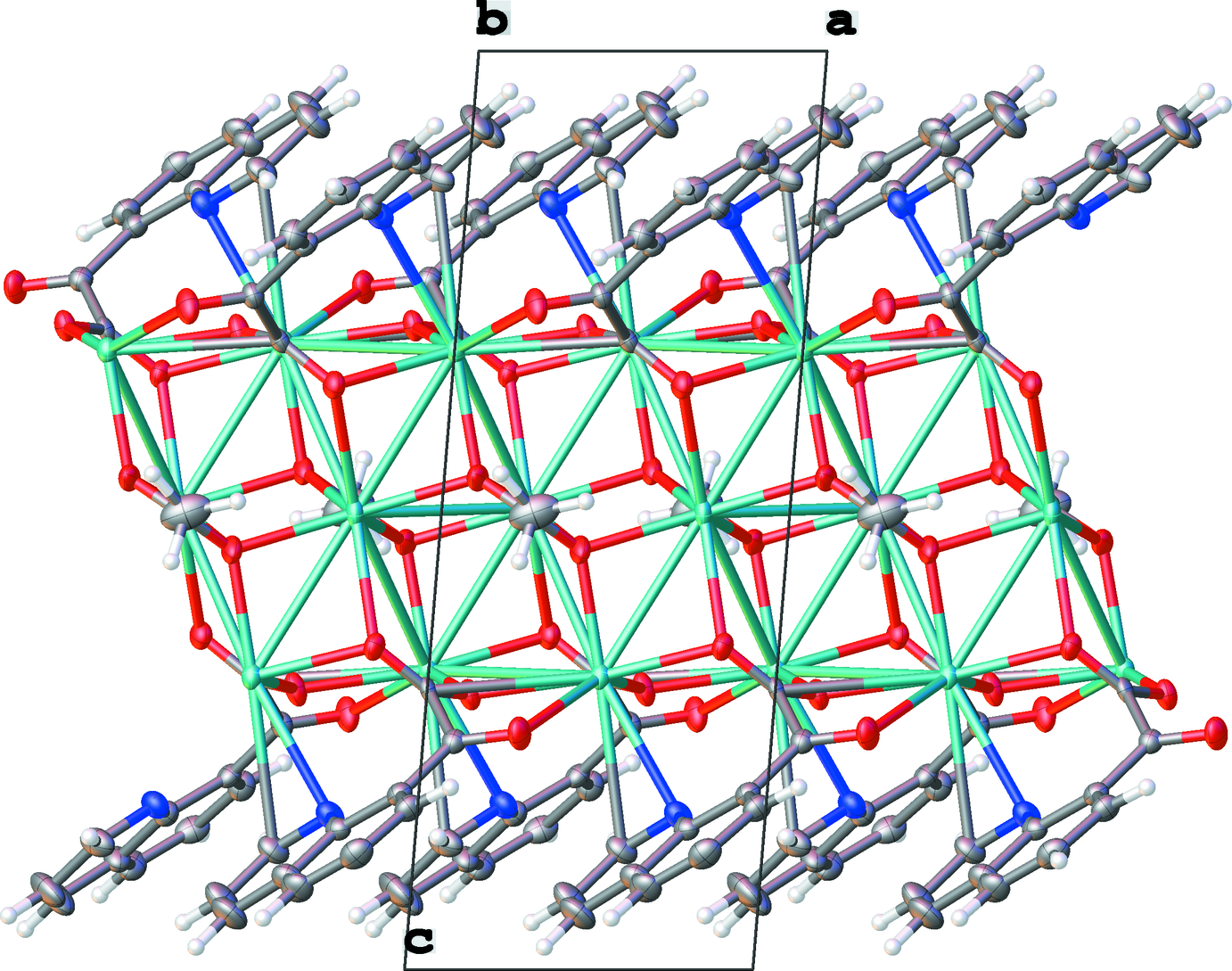

Supplement: Supplementary file 4 [file e-70-0m385-fig2.tif]

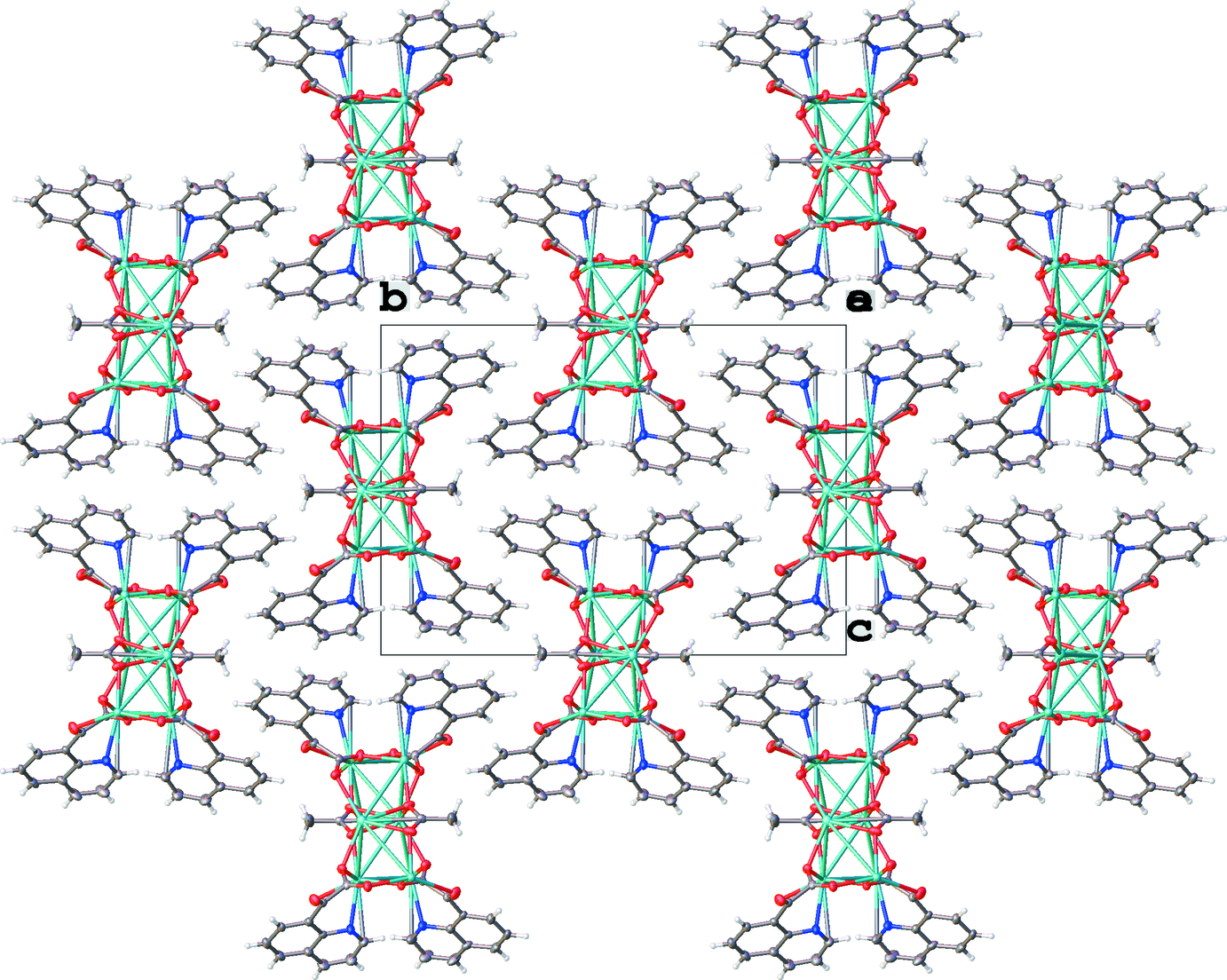

Supplement: Supplementary file 5 [file e-70-0m385-fig3.tif]
